# Immunomodulatory natural polysaccharide‐based nanoparticles for the treatment of neurodegenerative diseases

**DOI:** 10.1002/ibra.12199

**Published:** 2025-05-12

**Authors:** Leto‐Aikaterini Tziveleka, Mariafrancesca Cascione, Paolo Pellegrino, Annalisa Bianco, Stefano Leporatti, Valeria De Matteis

**Affiliations:** ^1^ Section of Pharmacognosy and Chemistry of Natural Products, Department of Pharmacy National & Kapodistrian University of Athens Athens Greece; ^2^ Department of Mathematics and Physics “Ennio De Giorgi” University of Salento Lecce Italy; ^3^ Institute for Microelectronics and Microsystems (IMM) Lecce Italy; ^4^ CNR Nanotec‐Istituto Di Nanotecnologia Lecce Italy; ^5^ Department of Experimental Medicine University of Salento, Centro Ecotekne Lecce Italy

**Keywords:** immunomodulation, nanoparticles, natural polysaccharides, neurodegenerative disorders

## Abstract

Polysaccharide‐based nanoparticles offer significant potential for the treatment of neurodegenerative diseases and the modulation of inflammatory responses in the central nervous system. These biopolymers, when derived from natural sources, possess inherent immunomodulatory properties, which can be leveraged to regulate immune activity, positioning them as promising candidates for both prophylactic and therapeutic strategies. Furthermore, when integrated with other materials, polysaccharides form nanocomposites with enhanced structural, physicochemical, and biological properties, making them highly versatile platforms for drug delivery in the central nervous system. This review provides a comprehensive analysis of polysaccharide‐based nanoparticles, focusing on their application in the treatment of three major neurodegenerative diseases: Alzheimer's disease, Parkinson's disease, and multiple sclerosis. Emphasis is placed on optimizing these nanomaterials for targeted drug delivery and immune modulation, underscoring their potential to improve therapeutic outcomes in neurodegenerative disorders. The review also examines the structural, chemical, and biological characteristics of key polysaccharides, and explores their innovative roles in combating neuroinflammation and neurodegeneration.

## Graphical abstract

Polysaccharide‐based nanoparticles offer versatile platforms for drug delivery and immune modulation in neurodegenerative diseases. This review highlights their potential in treating Alzheimer's, Parkinson's, and multiple sclerosis, focusing on targeted delivery and neuroinflammation. Structural and biological features of key polysaccharides are explored, showcasing innovative strategies for improved therapeutic outcomes.

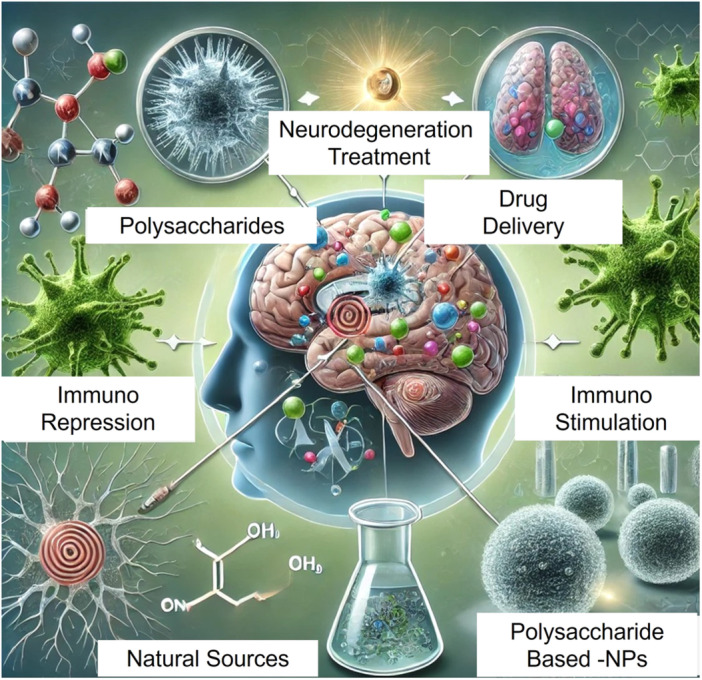

## 1 INTRODUCTION

The immune system is a sophisticated and well‐regulated defense mechanism distinguishing between self and non‐self. It is divided into two subsystems: innate and adaptive immunity. Innate immunity acts as the body's first line of defense, providing a nonspecific response through cells like macrophages and processes such as inflammation, coagulation, and complement activation. In contrast, adaptive immunity is antigen‐specific, generating stronger immune responses and immunological memory via T and B lymphocytes, which carry antigen‐specific receptors. Both subsystems work together synergistically to create a tailored immune response against external pathogens and internal disruptions.^1^ Given the critical role of the immune system in both health and disease, it has become a key target for therapeutic interventions. Immunomodulatory therapies aim to adjust immune responses by either enhancing or suppressing them, targeting a wide range of cells including dendritic cells, macrophages, T cells, B cells, and natural killer (NK) cells.^2^, ^3^, ^4^ These therapies hold significant potential in treating conditions where the immune system is either overactive, as in autoimmune disorders, or underactive, as in immunodeficiency and cancer.

The immune system plays a pivotal role in the development and progression of neurodegenerative diseases, including Alzheimer's disease (AD), Parkinson's disease (PD), and multiple sclerosis (MS). Chronic neuroinflammation, driven primarily by the activation of immune cells in the central nervous system (CNS) is a hallmark of these conditions.^5^ Under normal conditions, immune cells, such as microglia, act as the primary defenders within the CNS, clearing cellular debris and maintaining homeostasis. However, in neurodegenerative diseases, persistent activation of microglia leads to the release of pro‐inflammatory cytokines and reactive oxygen species, which exacerbate neuronal damage.^1^ Therefore, targeting the immune response within the CNS is increasingly recognized as a promising therapeutic approach for neurodegenerative diseases.^6^

Polymeric nanoparticles have emerged as innovative tools, capable of modulating the immune system by delivering therapeutic agents directly to affected areas. These nanoparticles can be engineered to cross the blood‐brain barrier (BBB) and transport anti‐inflammatory drugs or immunomodulatory compounds, offering a targeted strategy to regulate overactive immune cells and reduce harmful inflammatory pathways.^7^ By doing so, nanoparticle‐based therapies represent a frontier in addressing the immune‐mediated aspects of neurodegenerative disorders.^7^

Various polymeric nanomaterials have been developed as smart drug delivery vehicles to improve the pharmacokinetics and pharmacodynamics of toxic drugs, preserving their activity while minimizing side effects.^8^ Typically, pharmacologically inert polymers are used in drug delivery applications, but polymers with inherent pharmacological properties have been proposed for their added therapeutic benefits.^9^ The macromolecular nature of most biological targets in therapeutic interventions allows multivalent interactions that activate or inhibit signaling pathways. Due to their size and functional versatility, polymers of nanosized dimensions can replicate the multivalent features of macromolecules, enhancing their therapeutic efficacy compared to traditional small‐molecule drugs.^10^

Among these macromolecular biopolymers, natural polysaccharides are promising candidates for immune system modulation due to their inherent biocompatibility and ability to influence immune responses. When combined with other materials, these polysaccharides can form nanocomposites with tailored properties, enabling the development of efficient and biocompatible biomedical devices. Their immunomodulatory potential allows them to be used for a wide range of prophylactic and therapeutic purposes.^11^

In this context, natural polysaccharides show great promise in the design of immunomodulating drug delivery systems. Their structural and physicochemical properties are closely related to their biological activities, allowing for highly effective interactions with immune targets. Furthermore, by combining these polysaccharides with other polymers or active compounds, the creation of innovative biomaterials with enhanced therapeutic capabilities can be attained. This review provides an in‐depth and comprehensive analysis of polysaccharide‐based nanoparticles, with a focus on their application in treating three major neurodegenerative diseases: AD, PD, and MS. These conditions are characterized by progressive neuronal damage and chronic inflammation within the CNS, which pose significant challenges to conventional therapeutic approaches. Polysaccharide‐based nanoparticles offer a promising solution due to their biocompatibility, biodegradability, and inherent immunomodulatory properties. As such, they present a unique opportunity to develop targeted therapies that can effectively address both neurodegeneration and inflammation.

Particular emphasis is placed on optimizing these nanomaterials for targeted drug delivery, allowing for precise therapeutic interventions while reducing off‐target effects. In addition, we explore the structural, chemical, and biological properties of key polysaccharides, and how they can be fine‐tuned to enhance therapeutic outcomes. By focusing on immune modulation and neuroprotection, this review highlights the potential of polysaccharide‐based nanoparticles to revolutionize the treatment of CNS disorders. Moreover, it examines the molecular mechanisms through which these materials can reduce neuroinflammation and promote neuronal regeneration, providing a clear perspective on how integrating polysaccharides into nanotechnology could lead to novel and more effective treatments for neurodegenerative diseases.

## 2 NATURAL POLYSACCHARIDES: BIOPOLYMERS PRESENTING INTRINSIC IMMUNOMODULATORY PROPERTIES

**Figure 1:**
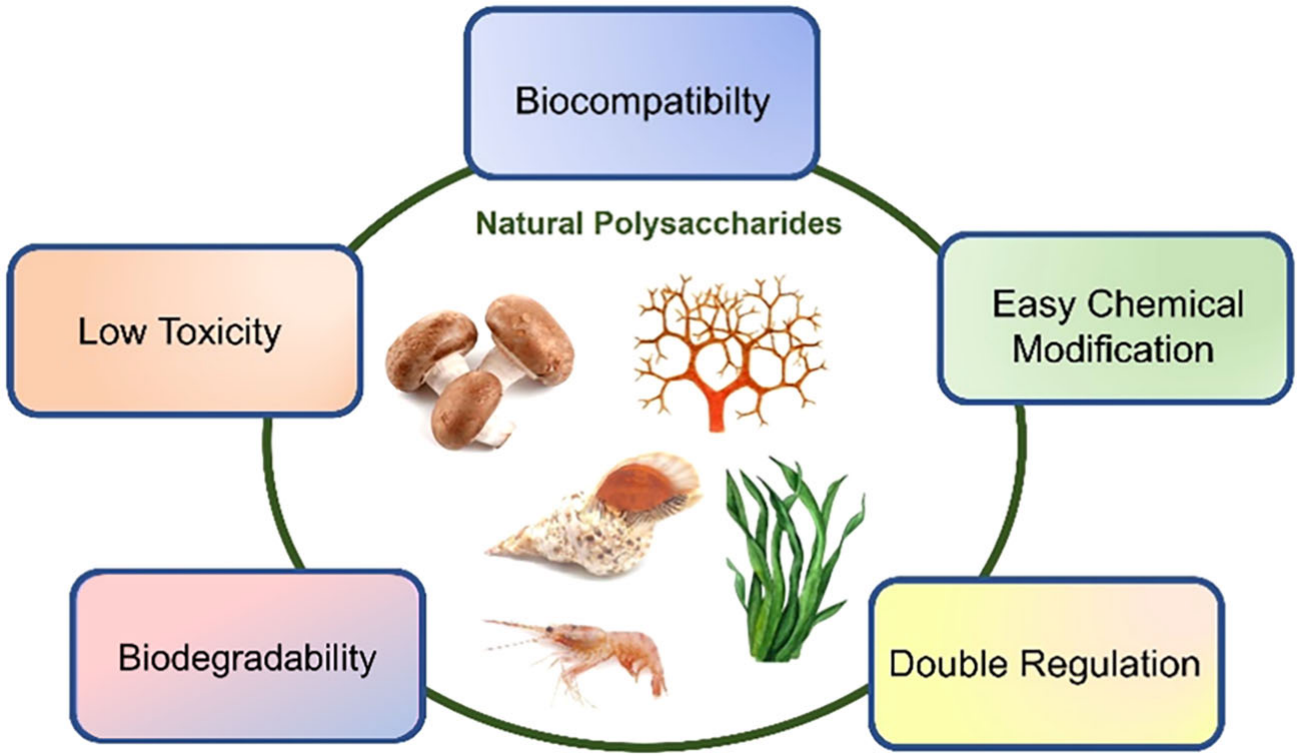
Advantageous characteristics of natural polysaccharides in bio‐applications. [Color figure can be viewed at wileyonlinelibrary.com]

Natural polysaccharides have emerged as pivotal components in the field of immunomodulation, due to their exceptional characteristics, but particularly due to their interactions with biological systems (Figure 1). These biopolymers are instrumental in shaping immune responses, which underscores their significance in therapeutic applications, especially concerning CNS disorders. Each category of polysaccharides possesses unique physicochemical properties that confer distinct advantages in modulating immune function, positioning them as promising candidates for addressing immune dysregulation in neurodegenerative diseases and other CNS‐related conditions. This chapter will delve into the potential of polysaccharides for immunoregulation within the CNS, emphasizing their physical and chemical characteristics. Furthermore, it will explore the underlying mechanisms of action and discuss their therapeutic implications.

### 2.1 The overview of natural polysaccharides

Natural polysaccharides are bioactive macromolecules derived from plants, animals, microorganisms, and algae, with over 300 types reported to date (Table 1).^12^ These polysaccharides consist of at least 10 monosaccharide units linked by various glycosidic bonds, forming either linear or branched long‐chain molecules. If composed of identical monosaccharide units, they are classified as homopolysaccharides, whereas when consisting of different units, they are referred to as heteropolysaccharides.

**Table 1:** Characteristic natural polysaccharides classified according to their origin (adapted from [12]).

Origin	Polysaccharides
Animals	chitin, chitosan, glycosaminoglycans (e.g., chondroitin, hyaluronic acid)
Plants	cellulose, glucans, glucomannan, hemicellulose, pectin, starch
Bacteria	bacterial cellulose, dextran, gellan, polygalactosamine, xanthan
Fungi	chitin, chitosan, pollulan, yeast glucans
Algae	agar, alginates, carrageenans, fucoidans, ulvans

Natural polysaccharides play various biological roles, such as energy storage (e.g., starch), providing structural support in plant cells (e.g., cellulose), forming the extracellular matrix (e.g., hyaluronic acid), and serving as ion carriers (e.g., alginate).^12^, ^13^, ^14^, ^15^, ^16^ They also have numerous health benefits, particularly for the immune system, exhibiting potent immunomodulatory properties.^17^, ^18^, ^19^ By targeting different cell signaling pathways, polysaccharides enhance immune cell activity, promoting cytokine secretion, which in turn activates macrophages and NK cells, leading to increased antigen phagocytosis. These properties make polysaccharides ideal candidates for immunomodulation.^12^, ^13^, ^14^, ^15^, ^16^, ^17^, ^18^, ^19^, ^20^

### 2.2 Physical properties of natural polysaccharides

Natural polysaccharides are macromolecules characterized by broad polydispersity, varying chain lengths, diverse molecular architectures (either linear or branched), and multiple reactive sites on each glycoside unit. These structures also contain various functional groups, such as acetyl and/or sulfate groups, which further contribute to their complexity. The unique properties of polysaccharides arise not merely from their individual monomeric units but are predominantly governed by their spatial conformation and molecular organization.^21^, ^22^, ^23^, ^24^

Polysaccharides, along with oligosaccharides and monosaccharides, are generally soluble in aqueous media. However, highly crystalline polysaccharides like cellulose, agarose, and starch are insoluble in aqueous media and require polar solvents, such as pyridine, dimethylformamide, or dimethyl sulfoxide, for dissolution.^25^ Their degree of crystallinity varies; for instance, sucrose and cellulose exhibit well‐defined crystalline structures,^26^ whereas other carbohydrates may exist in amorphous forms, such as glasses or solids, due to their conformational flexibility.

**Figure 2:**
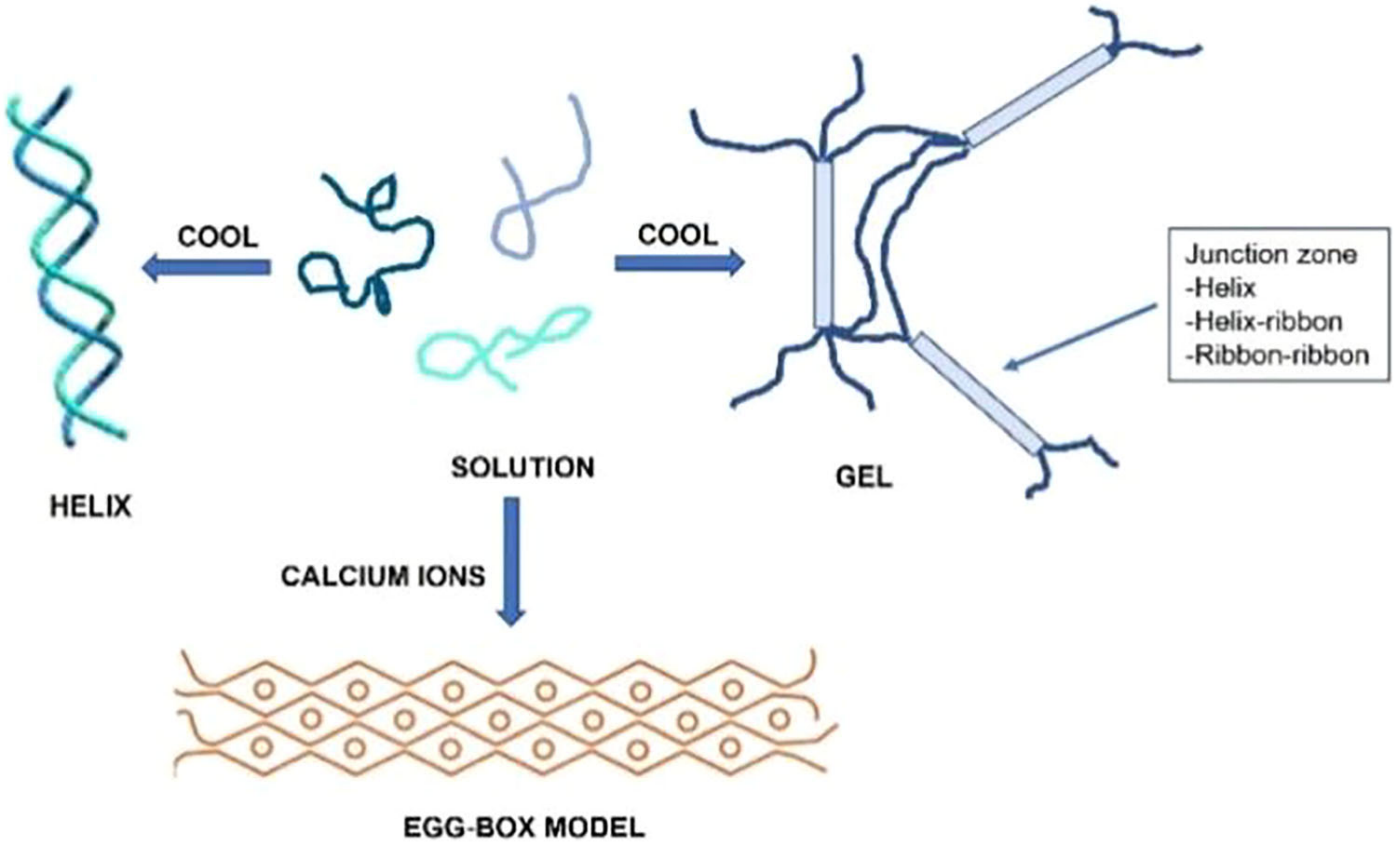
Formation of helix or gel from a polysaccharide dissolved in hot aqueous solution after cooling and in the presence of calcium ions. [Color figure can be viewed at wileyonlinelibrary.com]

Regarding their stability, polysaccharides are generally stable compounds, though reducing sugars are susceptible to oxidation because of their hemiacetal or aldehyde groups. Certain polysaccharides can adopt stable secondary structures, such as helices, which may be reversibly altered by temperature fluctuations, pH changes, or the introduction of denaturing agents.^27^ In contrast, complex structures of higher order—such as gels, fibers, and triple helices—undergo irreversible denaturation, with their stability heavily reliant on cross‐linking between polysaccharide chains, often facilitated by ions like Ca²⁺ (Figure 2).^28^

### 2.3 Structure‐immunomodulatory activity relationship

The immunomodulatory properties of polysaccharides are known to be distinctly manipulated by their monosaccharide composition, glycosidic bond types, molecular weight (*M*_W_), the presence of functional groups, branching, and chain conformation.^29^, ^30^, ^31^, ^32^ The complexity of the structural characteristics affects the biological properties in an inconsistent and even controversial manner. Moreover, differences in the natural source and extraction methods may result in structural, and therefore, bioactivity variations for the same class of polymers. Hence, strict protocols must be applied for the preparation of polysaccharide nanomaterials with defined morphological and physicochemical characteristics (e.g., shape, size, and stiffness).

Macrophages and dendritic cells, which express high levels of carbohydrate receptors, can specifically recognize saccharide structures, activating signaling pathways that lead to cellular responses and functional changes.^33^, ^34^ The immunological effects of polysaccharides are influenced by their chemical composition, particularly the monosaccharides they contain. For example, β‐glucans, composed of β‐(1 → 3)‐linked d‐glucose, demonstrate various immunomodulatory activities by regulating cytokines such as nitric oxide (NO), interleukins (ILs), tumor necrosis factor alpha (TNF‐α), interferon‐gamma (IFN‐γ), and non‐cytokine mediators like prostaglandin E2 (PGE2).^34^ A β‐glucan‐rich polysaccharide isolated from the mushroom *Echinodontium tinctorium* showed both immunostimulatory and anti‐inflammatory effects in macrophage and mouse models. Similarly, polysaccharides from *Hericium erinaceus* exhibited immunomodulatory activity, with β‐(1 → 3)‐branched‐β‐(1 → 6) glucan outperforming β‐(1 → 3)‐branched‐β‐(1 → 6) mannan in promoting NO synthesis and cytokine expression. Additionally, xylose‐rich heteroglycans from flaxseeds mediate immunostimulatory effects through toll‐like receptors (TLR2) and mitogen‐activated protein kinase (MAPK) pathways, while fucoidans, primarily composed of fucose, show anti‐inflammatory properties.^35^, ^36^, ^37^, ^38^, ^39^, ^40^, ^41^

The type and position of glycosidic linkages also affect the anti‐inflammatory activities of polysaccharides. Specifically, β‐(1 → 3), (1 → 6) glycosidic linkages have been demonstrated to significantly enhance the immunomodulatory effects of polysaccharides.^42^ β‐(1 → 3)‐Branched‐β‐(1 → 6)‐glucan, consisting of (1 → 6)‐linked glucopyranosyl backbone and a side chain composed of (1 → 3)‐linked glucopyranosyl, exerted significant macrophage activation.^36^ In another study, a β‐(1 → 3)‐ branched‐β‐(1 → 2)‐mannan possessing α‐(1 → 2)‐linked mannopyranosyl main chain and α‐(1 → 3)‐linked mannopyranosyl side chain could also activate macrophages.^37^ In other studies, the requirement of (1 → 3) linked α‐D glucose was reported for macrophage activation to occur.^43^ In a recent study, two polysaccharides obtained by solid‐state fermentation of the fungus *Fusarium solani* DO7 were shown to exert significant immunomodulatory activity, with the one lacking (1 → 2,6)‐ mannopyranosyl glycosidic linkages possessing the higher activity.^44^ The existence of side chains is also of prime importance since Tang et al. (2020) showed that partial removal of the side chains of arabinogalactans derived from *Larix principis‐rupprechtii* enhanced their immunomodulatory activity, while excessive removal resulted in its severe decrease.^45^

The sulfate content and *M*_W_ of polysaccharides significantly influence their biological activities. Sulfated polysaccharides exhibit stronger immunomodulatory effects than non‐sulfated ones by modulating MAPK and nuclear factor‐κB (NF‐κB) signaling pathways.^46^ For example, the depolymerization of ulvan reduced its immunomodulatory capacity in RAW264.7 macrophages.^47^ A study on sulfated polysaccharides from *Sargassum cristaefolium* found that a polysaccharide with a *M*_W_ of 386.1 kDa and 9.42% sulfate content had the highest NO inhibition in lipopolysaccharide (LPS)‐stimulated macrophages, suggesting that sulfate content alters cell receptor affinity, affecting NO production.^17^ Moreover, a Chinese yam polysaccharide after sulfate modification with no impact on the main chain structure, demonstrated improved immunomodulatory activity via viability increase of RAW 264.7 macrophage cells and stimulation of NO, TNF‐α and interleukin‐6 (IL‐6) production. The sulfated polysaccharide induced the activation of MAPK and NF‐κB pathways through TLR4.^48^ Nevertheless, the exact effect of each parameter is difficult to be distinguished, as in the case of a polysaccharide isolated from *Salvia miltiorrhiza* and its sulfated derivative, which both exerted strong immunomodulatory activity and regulated cytokines (IL‐2, IL‐6 and TNF‐α), but differed substantially in their *M*_W_ (1.28 × 10^3^ kDa and 81.7 kDa, respectively).^49^

**Figure 3:**
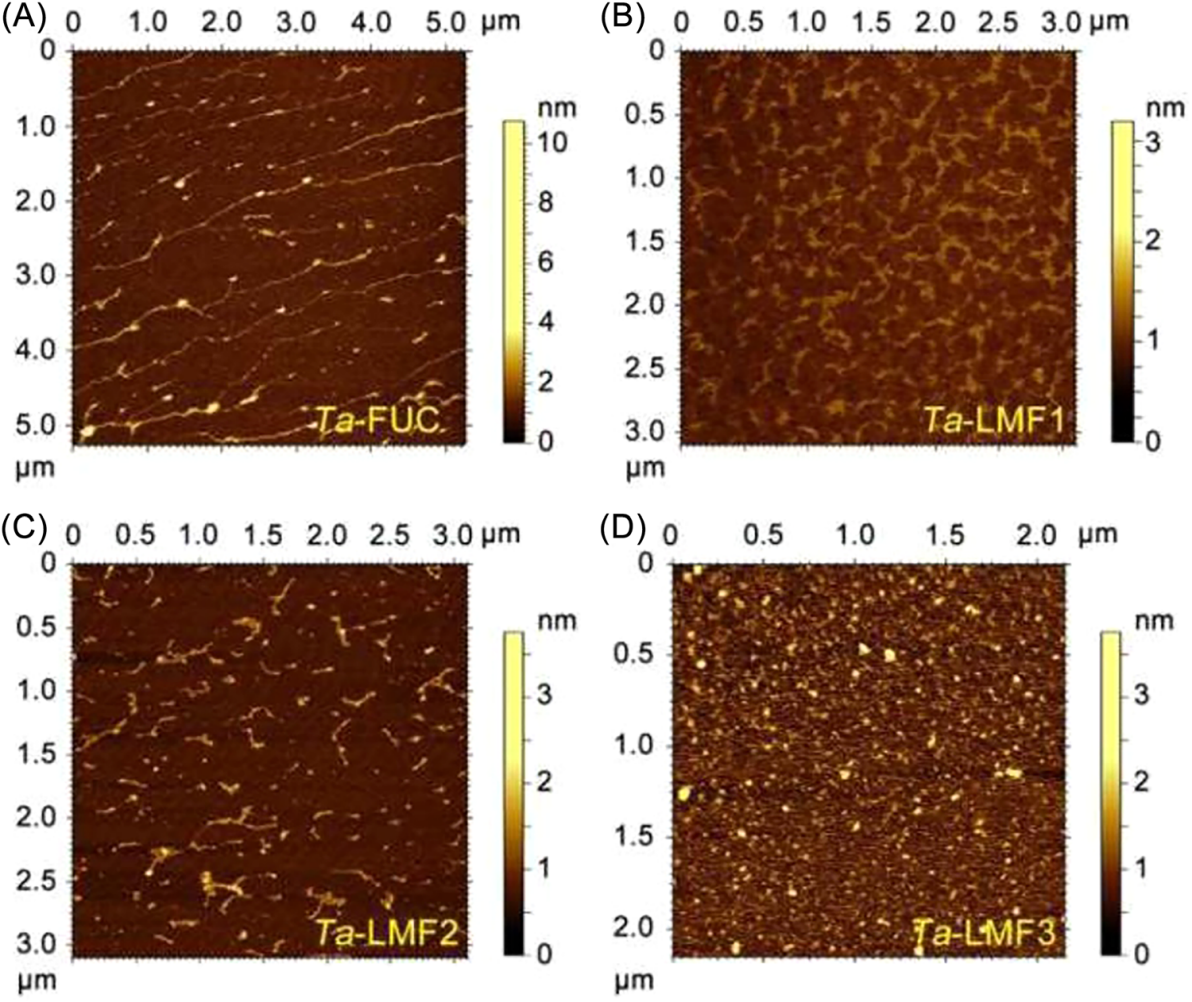
Atomic force microscopy (AFM) images of (A) fucoidan of high molecular‐weight (Ta‐FUC, _W_ 1380.0 kDa), (B) low‐molecular‐weight fucoidans (Ta‐LMF1, *M*_W_ 828.7 kDa), (C) Ta‐LMF2 (*M*_W_ 483.0 kDa) and (D) Ta‐LMF3 (*M*_W_ 215.0 kDa) (reproduced from [50]). [Color figure can be viewed at wileyonlinelibrary.com]

*M*_W_ variations can alter the chain conformation of polysaccharides, influencing immunomodulatory activity.^50^ In this framework, Xu et al. studied the gastric protective activities of sea cucumber fucoidans of varying *M*_W_ in an ethanol‐induced gastric ulcer model, involving antioxidative and anti‐inflammatory mechanisms.^50^ It was found that upon *M*_W_ decrease, the gastric protective effect decreased initially and subsequently recovered. Morphological studies revealed that fucoidan of high *M*_W_ (Ta‐FUC) adopted stiff random coil conformations which might act as a physical barrier outside the gastric mucosa blocking ethanol; a similar phenomenon has been observed in other polysaccharides such as pectin.^51^ As *M*_W_ declined, the chain stiffness and molecular size decreased gradually until the chain conformation transited from coil to a small‐sized sphere (Ta‐LMF3), a supposedly suitable structure for the penetration and diffusion in gastric tissue as well as the interaction with cellular receptors (Figure 3).^50^

Chain conformation appears to be the ultimate factor influencing the immunomodulatory activity of polysaccharides. Once in solution, polysaccharides acquire different conformations such as random coil, helical, including single and triple helix, rod‐like, and sphere‐like shapes, the triple‐helix state being considered as the most active one.^52^, ^53^ For example, β‐glucans that exhibit strong immunomodulatory activity, usually adopt the triple‐helix conformation. The better recognition of β‐glucans in the triple‐helix conformation by the receptors of immune cells because of higher stiffness was suggested as the main reason for their advanced immunomodulatory activity.^53^, ^54^

### 2.4 Immunomodulatory effects of selected polysaccharides

**Figure 4:**
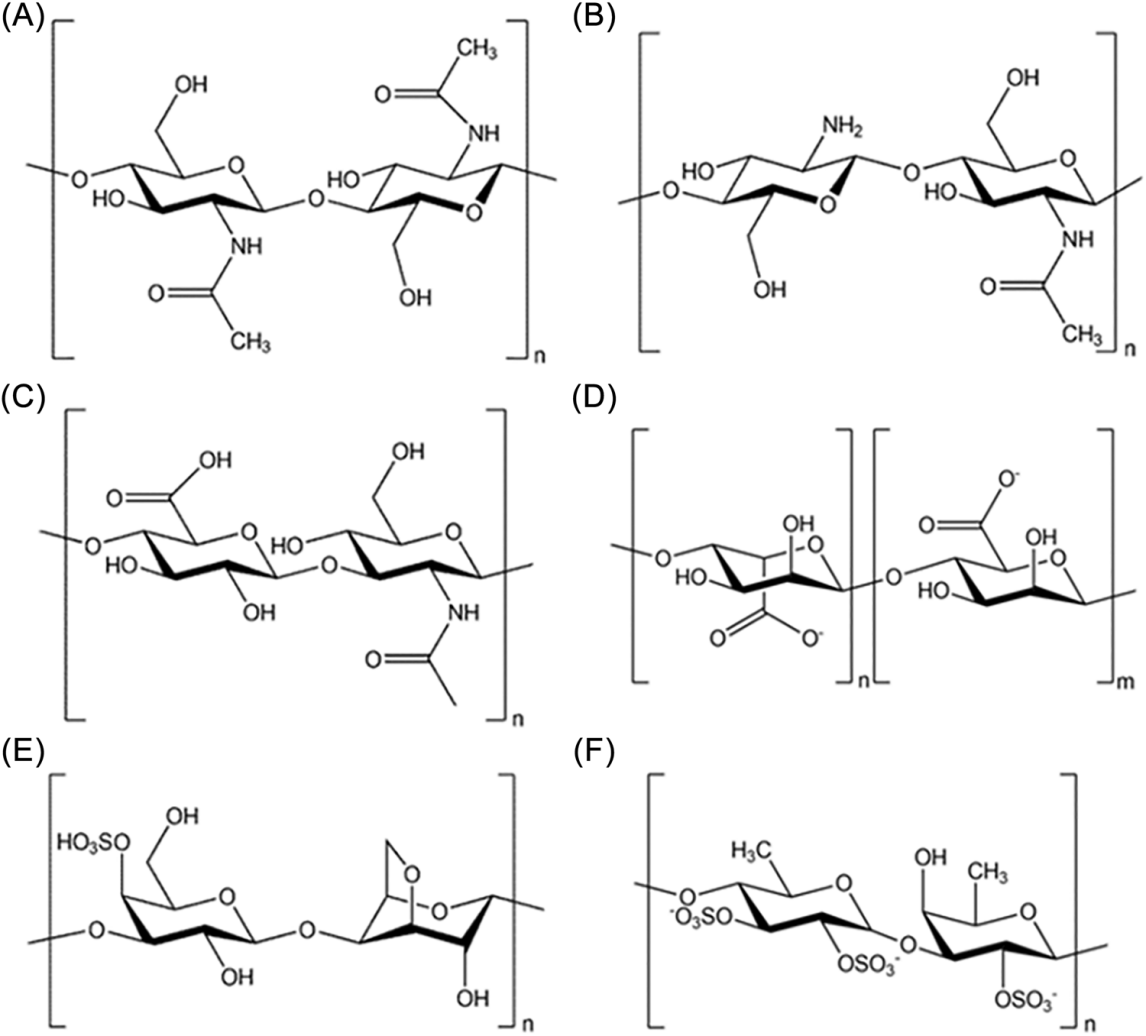
Schematic representation of (A) chitin, (B) chitosan, (C) hyaluronic acid, (D) alginate, (E) carrageenan, and (F) fucoidan macromolecules.

As discussed in the previous section, numerous natural polysaccharides have been extensively studied for their capacity to modulate both innate and adaptive immune responses through direct and indirect mechanisms. In this section, we will analyze the immunomodulatory effects of some of the most well‐studied and commercially available polysaccharides, such as chitin, chitosan, and hyaluronic acid, as well as polysaccharides derived from seaweeds, including alginate, carrageenans, and fucoidans. These polysaccharides will be examined for their functional behavior, which can influence the immune system in various ways. Special emphasis will be placed on their structural features, the specific mechanisms through which they modulate immune cell activity, and their involvement in neuroimmune interactions. The chemical structures of these polysaccharides are illustrated in Figure 4.

#### 2.4.1 Chitin and chitosan

Chitin, a hydrophobic linear biopolymer primarily derived from crustacean exoskeletons, consists of 2‐acetamido‐2‐deoxy‐d‐glucopyranose linked via β‐(1 → 4) bonds (β‐(1,4)‐*N*‐acetyl glucosamine) (Figure 4A).^55^ Chitin exhibits both pro‐inflammatory and anti‐inflammatory effects, influenced by its molecular size, shape, and purification method.^56^, ^57^ While larger chitin molecules (>70 µm) are inert, intermediate chitin (40–70 µm) and degraded small chitin molecules (<40 µm) have different effects. Specifically, intermediate chitin triggers pro‐inflammatory responses via TLR2 and NF‐κB, leading to TNF production, while small chitin promotes anti‐inflammatory responses by producing interleukin‐10 (IL‐10).^58^ For example, intermediate‐sized chitin fragments stimulate TNF‐α expression in macrophages via TLR2 and interleukin‐17A (IL‐17A)‐dependent pathways, while larger fragments remain inert.^59^ Chitin is also recognized as a pathogen‐associated molecular pattern (PAMP) by specific receptors, activating immune responses and cytokine expression, and enhancing T cell, NK cell, and IFN‐γ activity.^60^, ^61^, ^62^

In AD, activated microglia and astroglia release pro‐inflammatory cytokines, contributing to neuroinflammation. The neuroinflammatory hypothesis suggests that amyloid‐beta (Aβ)‐induced microglia activation and NLR family pyrin domain containing 3 (NLRP3) inflammasome activation leads to neurotoxicity.^63^ Microglia engulf chitin debris, and both microglia and neurons produce *N*‐acetylglucosamine polymers, which may worsen neurotoxicity. Accumulation of intermediate chitin in the brain could activate microglia, linking chitin deposits to neuroinflammation in AD.^64^, ^65^

Chitosan, the deacetylated derivative of chitin (Figure 4B), is a cationic polyelectrolyte that also modulates immune responses.^66^ It is commonly used to create brain targeting nanostructures that cross the BBB via adsorption‐mediated transcytosis, especially targeting neuronal cells in AD therapy.^67^ The immune effects of chitosan vary depending on its molecular form, with larger molecular weight chitosan being predominantly anti‐inflammatory and smaller forms promoting inflammation.^68^ Chitosan's immunomodulatory properties are associated with macrophage activation through TLR4 and inhibition of MAPK signaling, leading to reduced cytokine production.^69^, ^70^, ^71^, ^72^, ^73^ High molecular weight water‐soluble chitosan (WSC) has been shown to inhibit the production of pro‐inflammatory cytokines in an in vitro model for AD of human astrocytoma cells activated by Aβ peptide 25–35 (Aβ_25–35_) and interleukin‐1 beta (IL‐1β). WSC significantly reduced the secretion of pro‐inflammatory cytokines, such as TNF‐α and IL‐6, at concentrations of 1 or 10 μg/mL, while it also partially inhibited inducible nitric oxide synthase (iNOS) expression induced by Aβ_25–35_ and IL‐1β.^74^

#### 2.4.2 Hyaluronic acid

Hyaluronic acid (Figure 4C) is a linear polyanionic polysaccharide abundantly present in the extracellular matrix of vertebrate tissues, composed of a repeating disaccharide unit of (1 → 4)‐d‐glucuronic acid‐β‐(1 → 3)‐N‐acetyl‐d‐glucosamine.^16^ It belongs to the class of non‐sulfated glycosaminoglycans and exerts immunomodulatory properties most probably via the interactions with CD44 (a glycoprotein involved in cell adhesion and migration) which further regulates cytokines production.^75^ Hyaluronic acid of high *M*_W_ induces a size‐dependent inflammatory response regulation, demonstrating anti‐inflammatory and immunosuppressive properties, while when of low *M*_W_, it acts as a potent pro‐inflammatory molecule.^76^, ^77^, ^78^ Nevertheless, in another study, hyaluronic acid of varying *M*_W_ was reported to trigger stimulatory activity on diverse immune cells. Specifically, in a primary culture of rat microglial cells stimulated with LPS, the co‐treatment with high *M*_W_ hyaluronans reduced the production of IL‐1β and IL‐6. This reduction occurred through the inhibition of the extracellular signal‐regulated kinase‐1/2 (Erk 1/2) and Akt signaling pathways.^79^

#### 2.4.3 Alginates

Alginates (Figure 4D) are naturally occurring polysaccharides derived from brown algae, formed by alginic acid and its salts, such as sodium, ammonium, and calcium. Alginic acid is a linear macromolecule that consists of homopolymer blocks of (1 → 4)‐linked β‐d‐mannuronic acid (M) and (1 → 4)‐linked α‐l‐guluronic acid (G) residues, arranged in a homogenous (M‐blocks, G‐blocks) or heterogenous (M‐G‐blocks) block‐like pattern.^12^, ^80^ Alginates are known to modulate both innate and adaptive immune responses. They activate the innate immune system through NF‐κB signaling, leading to the production of pro‐inflammatory cytokines, including IL‐1β, IL‐6, IL‐12, and TNF‐α.^81^ In vitro and in vivo studies demonstrated that alginates, especially those with low viscosity or in particulate form, effectively stimulate cytokine production (IL‐1β, IL‐8, TNF‐α, IFN‐γ) in dendritic cells, macrophages, and splenocytes.^82^ Additionally, alginate oligomers induce cytokine secretion from human mononuclear cells, with M‐oligomers being more potent than G‐oligomers in stimulating TNF‐α production in RAW264.7 cells.^83^, ^84^ However, alginate shows promise as a nutraceutical or therapeutic agent for neurodegenerative diseases, particularly in AD. Alginate protects NT2 neurons against H_2_O_2_‐induced neurotoxicity by up regulation of heme oxygenase 1 (HO‐1), gamma‐glutamyl cysteine synthetase (γ‐GCS), heat shock protein 70 (HSP70), nuclear erythroid‐related factor‐2 (Nrf2) and inhibiting caspase‐3 and NF‐κB.^85^ In addition, alginate‐derived oligosaccharide reduced NO and PGE2 in BV2 microglia, lowering iNOS, cyclooxygenase‐2 (COX‐2), pro‐inflammatory cytokines, and TLR4/NF‐κB overexpression after LPS/Aβ stimulation.^86^

#### 2.4.4 Carrageenans

Carrageenans (Figure 4E) are high‐molecular‐weight sulfated polysaccharides derived from red seaweeds, composed of alternating units of d‐galactose and 3,6‐anhydro‐galactose linked by α‐(1 → 3) and β‐(1 → 4) glycosidic bonds, with sulfate content ranging from 15% to 40%.^87^ The primary forms—kappa, iota, and lambda—differ in sulfation degree and solubility. Carrageenans induce cell migration, plasma exudation, and inflammatory mediator production, including prostaglandins and NO via COX and iNOS activation.^88^ Their immunomodulatory effects depend on *M*_W_ and secondary structure.^89^, ^90^ Low *M*_W_ carrageenans (less than 20 kDa) demonstrate strong immunostimulatory properties, enhancing neutrophil phagocytosis, NK cell cytotoxicity, lymphocyte proliferation, and antibody‐dependent cell cytotoxicity.^90^ However, κ‐Carrageenan Oligosaccharide (KOS) exhibits immunomodulatory effects and may serve as a potential therapeutic intervention for neurodegenerative diseases related to inflammation. KOS significantly protects microglia from excessive inflammation by inhibiting the release of inflammatory cytokines and reducing oxidative stress.^91^ Additionally, KOS lowers the expression of proteins linked to the TLR4/NF‐κB and p38/JNK MAPK pathways that are activated by LPS in microglia.^92^

#### 2.4.5 Fucoidans

Fucoidans (Figure 4F), abundant in the brown algae cell wall, are sulfated polysaccharides composed mainly of l‐fucose.^93^ The polysaccharide backbone consists of repeating α‐(1 → 3) linked l‐fucopyranose residues or alternating α‐(1 → 3) and α‐(1 → 4) linked l‐fucopyranoses. The fucosyl residues can be sulfated and/or acetylated at various positions (on position C‐2 and C‐4 or rarely on C‐3).^94^ The chemical composition of fucoidans is highly variable, depending on the algal source, geographic location, the season of collection, and extraction method.^95^

Fucoidans are involved in various stages of the inflammatory process, including blocking lymphocyte adhesion, inhibiting enzymes, and inducing apoptosis. Their primary mechanism of action is through the downregulation of MAPK and NF‐κB signaling pathways, reducing pro‐inflammatory cytokine production.^96^ In macrophage studies, fucoidan inhibited NF‐κB activation, reduced TNF‐α and IL‐1β secretion, and prevented neutrophil infiltration, demonstrating its anti‐inflammatory potential.^97^ High *M*_W_ fucoidan further reduced NO, PGE2, TNF‐α, and IL‐6 production in LPS‐stimulated macrophages, while polysaccharides from the brown alga *Sargassum horneri* showed similar anti‐inflammatory effects.^98^, ^99^

A growing number of studies have shown that fucoidan also exerts a neuroprotective function. Cui et al. conducted both in vivo and in vitro studies demonstrating that fucoidan exerted neuroprotective effects on damaged dopaminergic neurons in a PD model induced by LPS. These effects were achieved by reducing intracellular ROS production, inhibiting the release of pro‐inflammatory cytokines, and decreasing the levels of TNF‐α.^100^

Microglial activation by LPS or Aβ is crucial in neurodegenerative disease development as it promotes pro‐inflammatory cytokines like IL‐1, TNF‐α, NO, and PGE2.^101^ Studies show that fucoidan can regulate this activation. For example, in a recent publication, the authors found that fucoidan inhibited NO and iNOS production in TNF‐α‐ and IFN‐γ‐stimulated C6 glioma cells and reduced LPS‐mediated microglial polarization.^102^ It also decreased pro‐inflammatory mediators, such as NO, PGE2, monocyte chemoattractant protein‐1 (MCP‐1), IL‐1β, IL‐8, and TNF‐α, in LPS‐stimulated microglia. Additionally, Choi et al. reported that fucoidan could modulate BBB permeability through its effects on selectin.^103^

## 3 IMMUNOMODULATORY POLYSACCHARIDE‐BASED NANOPARTICLES IN NEURODEGENERATIVE DISEASES

### 3.1 Complex immunomodulatory architectures based on polysaccharide‐derived nanoparticles

Complex architectures of polysaccharide‐based nanoparticles can be used to regulate the immunomodulatory response of functional drug delivery platforms. Stimuli responsiveness, multifunctionality, targeting ability, as well as multiple components can ensure the successful preparation of immunomodulatory materials.^104^

Polysaccharide‐based nanoparticles, typically ranging from 1 to 1000 nm, have emerged as promising drug delivery systems for treating neurodegenerative diseases and modulating CNS inflammation. Their biodegradable nature provides key advantages such as low toxicity, tunable degradation rates, high drug‐loading capacity, and the ability to cross the BBB, enabling more efficient and targeted delivery to the CNS compared to traditional polymer formulations.^105^ This enables more efficient and targeted delivery to the CNS compared to non‐nanostructured polymer formulations. A major challenge for anti‐inflammatory drugs is their inability to efficiently cross the BBB, limiting their access to target cells within the CNS, such as microglia and astrocytes.^106^ The BBB, a highly selective barrier composed of endothelial cells supported by astrocytes and pericytes, plays a crucial role in maintaining CNS homeostasis by preventing harmful substances like toxins and pathogens from entering. However, its restrictive properties also hinder the passage of most therapeutic agents, complicating treatment for neurological disorders.^107^, ^108^

To address these challenges, both invasive and noninvasive strategies have been developed to enhance drug delivery across the BBB. Among these, nanoparticle‐based systems are particularly promising, as they improve the stability and solubility of drugs while enhancing their transport across the barrier, increasing therapeutic efficacy.^109^, ^110^ This is particularly relevant for diseases such as AD, PD, Huntington's, frontotemporal dementia, and Lewy body dementia, which are characterized by neuroinflammation, protein aggregate accumulation, synaptic dysfunction, and progressive neuronal loss. These disorders lead to cognitive decline, memory impairments, and motor dysfunction, significantly reducing quality of life and contributing to a rising global burden.

Neuroinflammation, a hallmark of neurodegenerative diseases, is driven by hyperactivated microglia and astrocytes that release pro‐inflammatory cytokines like IL‐1, TNF‐α, and IL‐6, exacerbating neuronal damage.^111^, ^112^ This activation is often triggered by environmental toxins, pathogens, and protein aggregates such as Aβ, Tau, and α‐synuclein.^113^, ^114^ Consequently, therapeutic strategies aim to modulate neuroimmune responses, reduce inflammation, and enhance BBB penetration while addressing the complex cellular interactions underlying disease progression. By targeting microglia and astrocyte pathways, researchers hope to develop interventions that mitigate neuroinflammation and protect neurons, paving the way for more effective treatments.^115^, ^116^

### 3.2 Polymeric nanoparticles for AD

AD is a progressive neurodegenerative disorder primarily characterized by the accumulation of Aβ plaques and tau protein tangles, which result in synaptic dysfunction, neuronal loss, and cognitive decline. A critical aspect of AD pathology is neuroinflammation, which is largely mediated by the activation of microglia, the resident immune cells of the CNS. Microglia, in a healthy brain, are responsible for clearing debris and maintaining homeostasis. However, in AD, chronic activation of these cells leads to the release of pro‐inflammatory cytokines such as TNF‐α and IL‐1β, as well as ROS, all of which exacerbate neuronal damage and accelerate disease progression.^101^ Polymeric nanoparticles made from biocompatible polymers like poly(lactic‐co‐glycolic acid) (PLGA) and chitosan offer a promising solution for delivering therapeutic agents directly to sites of inflammation in the brain. Recent studies demonstrate that functionalizing these nanoparticles with ligands, such as transferrin or antibodies targeting activated microglia, can enhance their ability to penetrate the BBB and reach inflamed areas.^116^

Chitosan nanoparticles have emerged as a promising platform for direct nose‐to‐brain (N‐to‐B) drug delivery.^117^ Their potential is largely attributed to several key features that make them well‐suited for overcoming the challenges posed by the BBB and enhancing drug delivery to the brain. The cationic nature of chitosan allows interactions with the negatively charged surface of the BBB endothelial cells, facilitating easier penetration across the barrier, as well as with mucosal tissues, improving their retention in the nasal cavity and facilitating uptake. Furthermore, they exhibit strong mucoadhesive properties, allowing for greater delivery to the brain, and provide controlled drug release, which is essential for sustained therapeutic effects. The targeting ability of chitosan nanoparticles toward CNS is manipulated by adsorptive‐mediated transcytosis.^118^ Elnaggar et al. have successfully utilized chitosan nanoparticles to deliver piperine (PIP), a potent neuroprotective compound for AD. They developed targeted intranasal chitosan nanoparticles that efficiently penetrated the brain, showcasing exceptional entrapment efficiency and stability. In AD‐induced rats, nanoparticles that deliver PIP significantly enhanced cognitive function, comparable to the standard drug, donepezil, while providing additional benefits like acetylcholinesterase inhibition and antioxidant effects. Notably, the chitosan nanoparticles caused minimal nasal irritation, showed no brain toxicity, and demonstrated strong antiapoptotic and anti‐inflammatory properties. This approach reduced the necessary PIP dose by 20‐fold, highlighting its potential in AD treatment. Additionally, studies indicated that these nanoparticles led to significant reductions in neuroinflammation and improved cognitive performance, with nanoparticles demonstrating marked memory improvements and decreased neuroinflammatory markers.^119^

A hyaluronic acid‐based nanocarrier has been also developed for the direct N‐to‐B delivery of RNA for potential use in the treatment of neurological disorders.^120^ Initially, nanocomplexes between a hydrophobic derivative of octaargnine, a cell penetrating peptide, and RNA were formed via electrostatic interactions, and subsequently were enveloped in hyaluronic acid or a synthetic biopolymer. The prepared nanocarriers were able to overcome significant obstacles in N‐to‐B delivery, and increased the levels of therapeutic miRNA in hippocampus of AD mouse model, a brain area critical for learning and memory, resulting in the improvement of its function.

Alginate‐derived oligosaccharide (AdO) has been shown to significantly reduce levels of NO, PGE2, and other pro‐inflammatory cytokines. Additionally, AdO effectively diminished the overexpression of TLR4 and NF‐κB induced by LPS in BV2 cells.^86^ Furthermore, alginate micro‐encapsulation of mesenchymal stromal cells has been found to modulate the neuroinflammatory response by reducing PGE2 production in LPS‐stimulated astrocytes and microglia.^121^

**Figure 5:**
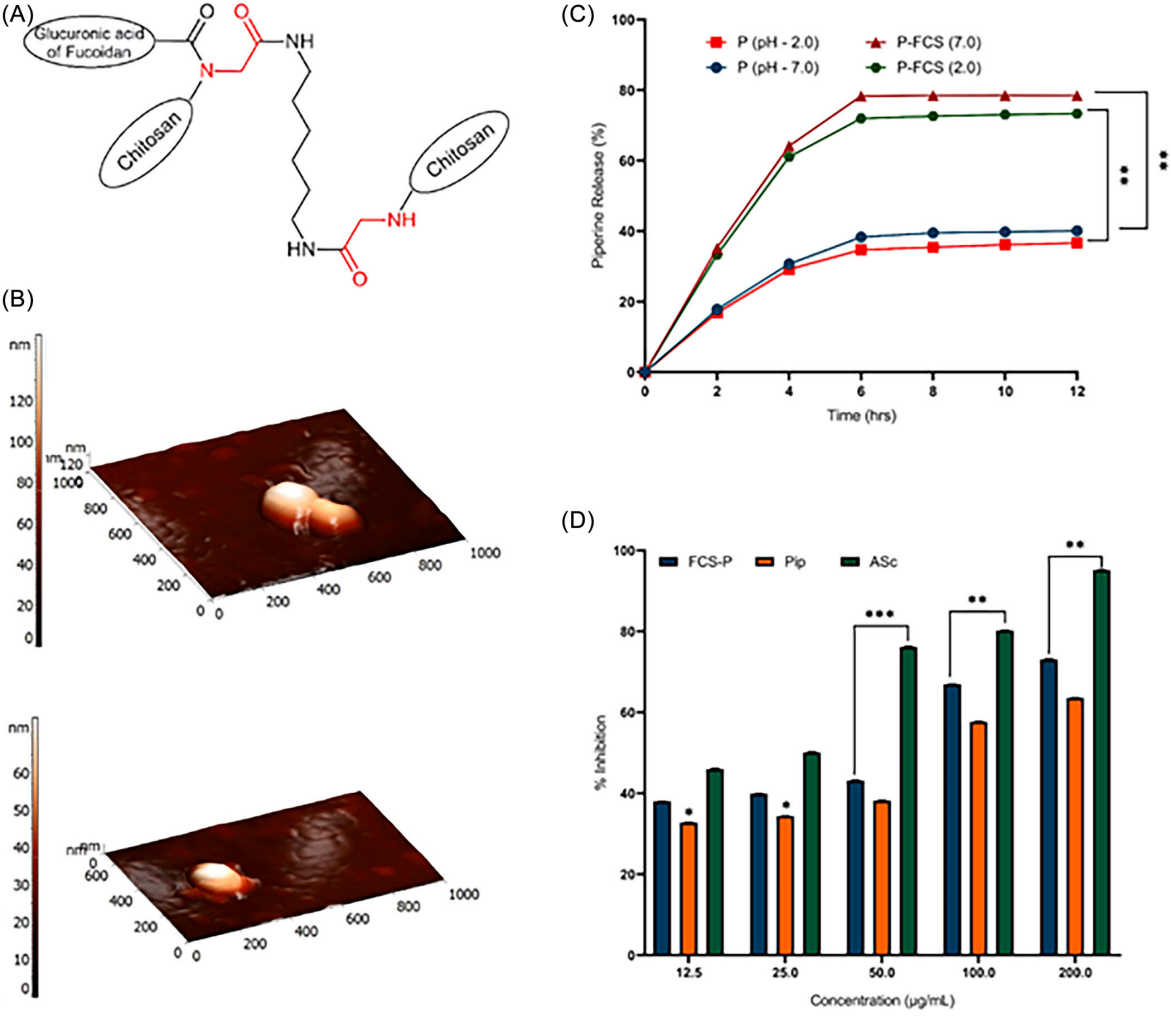
(A) Schematic representation of chemically crosslinked fucoidan‐chitosan particles. (B) 3D AFM images of the empty (upper part) and piperine‐loaded nanoparticles (lower part). (C) In vitro release of piperine from piperine‐loaded nanoparticles (P‐FCS) and unformulated piperine (P) in different pHs (*n* = 3). Statistical differences are shown for the piperine‐loaded nanoparticles and the unformulated piperine, **p < 0.01. (D) 2,2‐diphenyl‐1‐picrylhydrazyl (DPPH) scavenging activity of P‐FCS, P, and ascorbic acid (*n* = 3). *p*‐values for significantly different mean values (P‐FCS and P), *p < 0.05, **p < 0.01, and ***p < 0.001 versus ascorbic acid (i.e., control). (Reproduced from [124]). [Color figure can be viewed at wileyonlinelibrary.com]

Fucoidans have also been employed for the preparation of therapeutic nanoparticles for brain disorders, due to their implications in neuroprotective mechanisms.^122^ Their reported effects include the regulation of lipid metabolism, often mentioned in neurodegenerative diseases; the improvement of cholinergic activity, which is crucial for cognitive function and declines in AD; the maintenance of BBB integrity; the protection of mitochondria, thereby reducing the risk of neuronal damage; the reduction of oxidative stress, which contributes to cellular damage in the CNS; and finally, the inhibition of apoptotic pathways, slowing down the neuronal degeneration process.^123^ Fucoidan–chitosan particles loaded with PIP have been also prepared, exhibiting high encapsulation efficiency, controlled release profiles and high scavenging activity.^124^ Moreover, the activity of the PIP‐loaded particles was significantly higher than that of the free PIP, pointing to the synergistic effect of fucoidan and PIP (Figure 5). Fucoidan has also been used for the preparation of self‐assembled ovalbumin‐fucoidan nanoparticles loaded with nicotinamide mononucleotide, recognized as a promising compound in alleviating aging‐related mitochondrial dysfunction.^125^ The ovalbumin‐fucoidan nanoparticles reduced the oxidative stress and inhibited cellular senescence, indicating their potential use in age‐related diseases.

### 3.3 Polymeric nanoparticles for PD

PD is a neurodegenerative disorder characterized by the progressive loss of dopaminergic neurons in the substantia nigra, leading to motor and cognitive impairments. A key feature of PD is neuroinflammation, where immune cells in CNS, such as microglia and astrocytes, release inflammatory mediators like interleukins, TNF‐α, and ROS.^126^ This inflammatory response can be toxic to neurons, with excessive microglial activation contributing to the release of pro‐inflammatory cytokines and apoptosis, ultimately resulting in the degeneration of dopaminergic neurons. Thus, neuroinflammation plays a crucial role in the progression of PD and the severity of its symptoms.

It has been observed that chitosan nanoparticles loaded with curcumin can enhance neuroprotection by reducing oxidative stress, inhibiting inflammation, and promoting mitochondrial function. These nanoparticles effectively deliver curcumin to target cells, improving its bioavailability and stability, which in turn amplifies its therapeutic effects. This formulation has shown promise in protecting neurons from degeneration, supporting its potential use in treating neurodegenerative diseases.^127^

Clementino et al. examined the anti‐inflammatory properties of lecithin/chitosan nanoparticles loaded with statins, highlighting their potential for N‐to‐B delivery in treating AD and PD. The nanoparticles were found to effectively encapsulate statins, enhancing their stability and bioavailability. In vitro studies demonstrated that the statin‐loaded nanoparticles reduced inflammatory responses in neural cells by lowering levels of key inflammatory players such as TNF‐α, IL‐1β, and PGE2.^128^ Sridhar et al. demonstrated that encapsulating selegiline in a chitosan nanocarrier results in significant improvements in locomotor activity, catalepsy, and stride length in treated animals. This formulation not only enhances catalase activity but also increases levels of dopamine and glutathione in the brain while reducing neuroinflammatory players such as TNF‐α and IL‐1β, indicating a potential dual action of the treatment in both improving motor functions and mitigating neuroinflammation.^129^ Raj and colleagues showed that nasal formulations of pramipexole‐loaded chitosan nanoparticles significantly improved photoactometer scores and reduced motor deficits compared to pramipexole alone. These nanoparticles also elevated dopamine levels in the brain, enhanced antioxidant status, and decreased neuroinflammatory markers such as TNF‐α and IL‐1β. This indicates that the nanoparticles may enhance motor function while also mitigating neuroinflammation associated with PD.^130^ Recently, chitosan‐alginate nanoparticles have been synthesized and optimized for use as nonviral vectors for gene delivery, specifically for the transfection of a plasmid encoding Smad4. This advancement is significant, as Smad4 plays a crucial role in modulating inflammatory responses, which are particularly relevant in the context of PD.^131^

In another research, mild and limited mitochondrial damage, one of the primary causes of neuronal cell death in PD, was prevented by employing hyaluronic acid‐based nanoparticles.^132^ The protective role of hyaluronic acid against oxidative stress cellular damage through a mitochondrial‐controlled pathway, renders it a potent biopolymer for the development of nanoparticle systems for PD treatment.^133^, ^134^ In cases of irreversibly damaged mitochondria, addition of PTEN induced putative kinase 1 (PINK1) antibodies, targeting PINK1 protein known to accumulate intracellularly in damaged mitochondria,^135^ on hyaluronic acid‐based nanoparticles carrying a siRNA able to activate mitophagy in PD models, could promote the clearance of damaged mitochondria.

**Figure 6:**
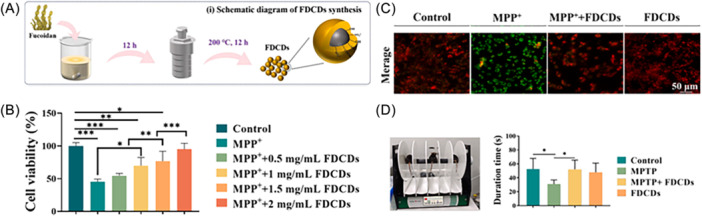
(A) Schematic diagram of FDCDs preparation. (B) Relative cell viability (*n* = 5) of MPP^+^‐treated cells in the presence and absence of different FDCDs concentrations. (C) Fluorescence images of mitochondrial membrane potential in MPP^+^‐treated cells. (D) Capacity of FDCDs to penetrate the BBB for PD treatment as evidenced by rotarod behavioral analysis (*n* = 6). FDCDs, fucoidan‐derived carbon dots; MPP^+^, 1‐methyl‐4‐phenylpyridinium ion; MPTP, 1‐methyl‐4‐phenyl‐1,2,3,6‐tetrahydropyridine (Reproduced from [136]). [Color figure can be viewed at wileyonlinelibrary.com]

Fucoidan‐derived carbon dots (FDCDs) have been recently proposed as nanopenetrants of BBB. The obtained fucoidan‐derived carbon dots retained all the key characteristics of carbon dots, as well as the presence of sulfate groups deriving from fucoidan, thus contributing to their interaction with biological systems due to their negatively charged surface.^136^ These nanoparticles demonstrated in vitro anti‐inflammatory, antioxidant, and antiapoptotic properties in 1‐methyl‐4‐phenylpyridinium ion (MPP^+^)‐induced damage in PC12 cells (MPP^+^ is the conversion derivative of neurotoxin 1‐methyl‐4‐phenyl‐1,2,3,6‐tetrahydropyridine (MPTP) that can damage neurons in the brain, causing symptoms similar to PD), while in vivo studies confirmed their ability to cross the BBB. Furthermore, intravenous administration of FDCDs into MPTP‐induced PD mice was effective in restoring motor function, suggesting that these carbon dots could be a promising treatment for PD (Figure 6).

### 3.4 Polymeric nanoparticles for MS

MS is a chronic, inflammatory, and neurodegenerative disease that directly impacts the CNS, causing immune‐mediated demyelination and axonal damage. The hallmark of this disease is a robust immune response, wherein peripheral immune cells infiltrate the CNS and release critical pro‐inflammatory cytokines, including IFN‐γ, TNF‐α and IL‐1β.^137^ These cytokines activate pathways that disrupt the BBB, permitting further immune cell entry into the CNS, which significantly amplifies the inflammatory response. This cascade activates microglia and macrophages, leading to the degradation of the myelin sheath and exacerbating neurodegeneration.^138^ In the progressive stages of MS, there is chronic activation of inflammatory pathways—especially those involving NF‐κB and Janus kinase/signal transducer and activator of the transcription signaling—which results in sustained neuroinflammation. This condition triggers oxidative stress, mitochondrial dysfunction, and excitotoxicity, inflicting widespread damage to neurons and glial cells. Consequently, demyelination and neurodegeneration accelerate, resulting in substantial cognitive decline and motor impairment.^139^ It is essential to recognize the pivotal role of inflammation in MS pathology. Therefore, therapeutic strategies that target these molecular pathways to modulate the immune response and diminish inflammation are critically important. Moreover, there is compelling evidence that sex hormones, such as estrogen and progesterone, significantly influence the immune response in MS, which contributes to the variations in disease course between men and women. A thorough understanding of these mechanisms will pave the way for more personalized and effective treatment approaches.^140^

**Figure 7:**
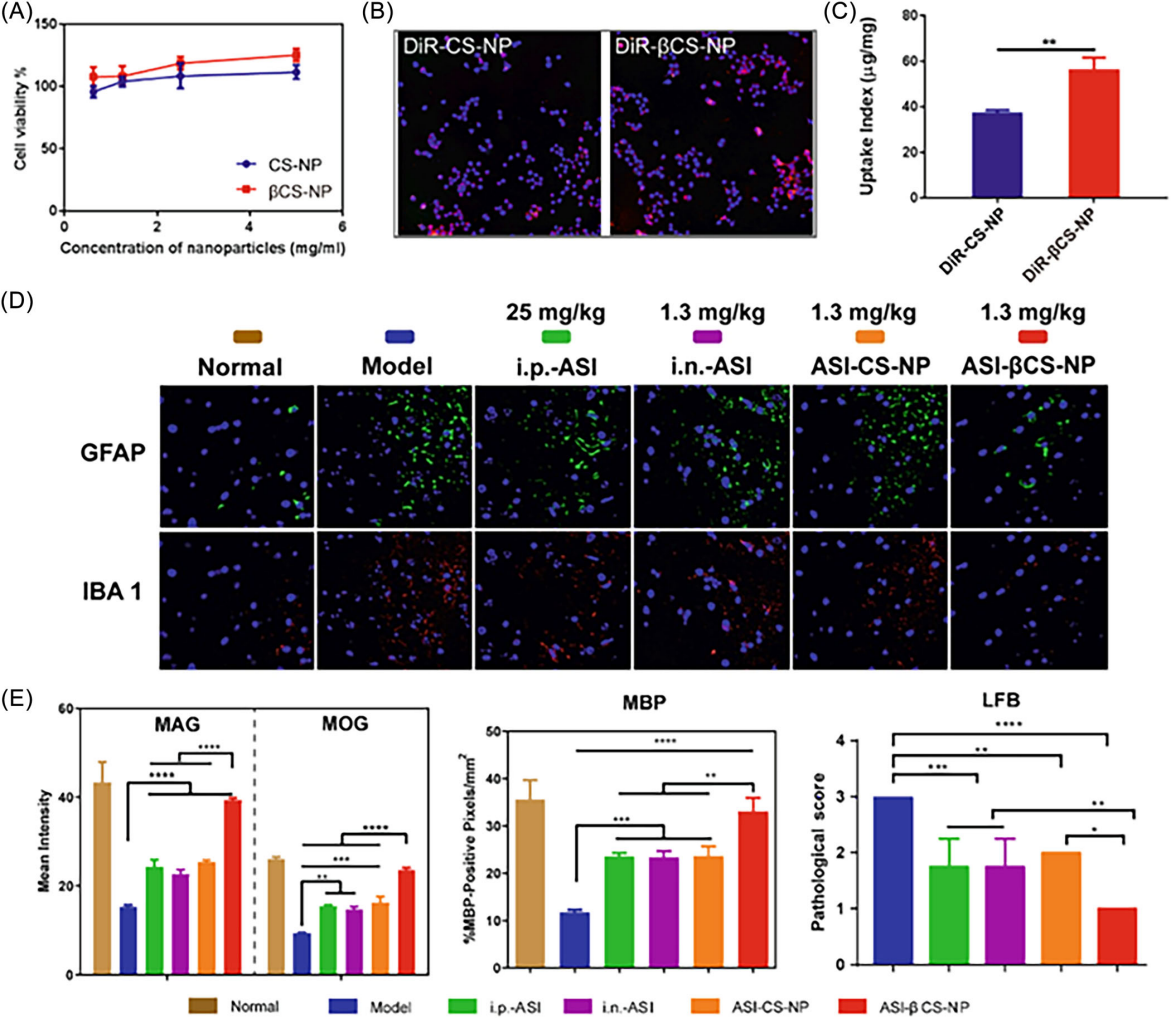
Brain targeting capacity of βCS‐NP as depicted by (A) cell toxicity of CS‐NP and βCS‐NP, (B) representative high‐content screening images of fluorescence signal after treatment with DiR with concentration of 10 μg/mL in 16HBE cells. Blue: 4′,6‐diamidino‐2‐phenylindole (DAPI), Red: DiR. (4× fields) and (C) the quantitative results of the DiR signal in 16 HBE cells presented as uptake index. (D) Immunofluorescence images of GFAP and IBA1 expression in the brain after intranasal (i.n.) administration of β‐asarone modified Astragaloside IV loaded chitosan nanoparticles (ASI‐βCS‐NP) in a mice experimental autoimmune encephalomyelitis (EAE) model showing significantly suppressed inflammatory infiltration and astrocyte/microglial activation (GFAP labeled green, IBA 1 labeled red, representative images of each group in 20× magnification). (E) Quantification results of the MAG, MOG, MBP expression, and LFB scores showing the reduced demyelination and increased remyelination on a mice EAE model after i.n. administration of ASI‐βCS‐NP (*n* = 5). CS‐NP, blank chitosan nanoparticles; βCS‐NP, β‐asarone modified chitosan nanoparticles; DiR, 1,1‐dioctadecyl‐3,3,3,3‐tetramethylindotricarbocyaine iodide; i.n., intranasal administration; i.p., intraperitoneal administration; GFAP, glial fibrillary acidic protein; IBA1, ionized calcium binding adaptor molecule 1; ASI, Astragaloside IV; MAG, myelin associated glycoprotein; MOG, myelin oligodendrocyte glycoprotein; MBP, myelin basic protein; LFB, Luxol fast blue (Reproduced from [142]). [Color figure can be viewed at wileyonlinelibrary.com]

Nanomedicines play a significant role in regulating immune cells involved in MS, such as dendritic cells, macrophages, and T‐cells. For instance, Pei et al. developed PLGA nanoparticles that encapsulate transforming growth factor‐β1 (TGF‐β1). When injected into a mouse model of experimental autoimmune encephalomyelitis (EAE), these nanoparticles increased the activity of regulatory T‐cells, inhibited the proliferation of T helper (Th)1, Th‐17, and cytotoxic T cells (Tc)‐1/Tc‐17 cells, and induced apoptosis in these cells. Additionally, elevated levels of TGF‐β1 and IL‐10 in the CNS and spleen demonstrated the potential of these nanoparticles to modulate autoimmune T‐cells.^141^ The β‐asarone modified Astragaloside IV (ASI) loaded chitosan nanoparticles (ASI‐βCS‐NP), were developed for nasal administration to treat MS to enhance the N‐to‐B delivery and therapeutic effects of ASI.^142^ ASI‐βCS‐NP significantly reduced behavioral scores, decreased weight loss, suppressed inflammatory infiltration and astrocyte/microglial activation, reduced demyelination, and increased remyelination in a mice EAE model (Figure 7). In another study by Li et al., polyethylene glycol (PEG)‐based nanoparticles were utilized to deliver a CRISPR‐Cas9 system targeting B220.^143^ These nanoparticles reduced the expression of B‐cell activating factor receptors, thereby inhibiting B‐cell proliferation, function, and survival. This disruption in B‐cell activity also impaired antigen presentation and plasma cell maturation, which are key processes in autoimmune conditions. These findings suggest that this approach could be effective in treating diseases related to B‐cell dysfunction, including MS.

In recent decades, gene therapy approaches for treating MS have garnered significant attention. In the context of nanomedicine, researchers have explored various methods for delivering genetic material to cells, including genomic DNA, plasmid DNA, siRNA, and oligonucleotides, all encapsulated within nanoparticles. For example, Kong et al. recently developed a chitosan‐based nanocarrier designed to improve the transfection efficiency of astrocytes, highlighting the potential for utilizing nanocarriers that combine active molecules with genetic material.^144^

## 4 CONCLUSIONS

The role of the immune system in the progression of neurodegenerative diseases, such as AD, PD, and MS, has become a critical focus for therapeutic interventions. Chronic neuroinflammation, driven by the activation of immune cells like microglia, is a central feature of these diseases, contributing significantly to neuronal damage and disease progression. Given the intricate relationship between the immune system and neurodegeneration, the modulation of immune responses within the CNS is emerging as a promising therapeutic approach. Polysaccharide‐based nanoparticles, derived from natural biopolymers, offer considerable potential in this context due to their biocompatibility, biodegradability, and inherent immunomodulatory properties. These nanoparticles can be engineered to cross the BBB, deliver therapeutic agents directly to the CNS, and regulate the overactive immune responses that drive neuroinflammation. Their ability to act as targeted drug delivery vehicles opens new avenues for precise, localized therapies that reduce off‐target effects and improve therapeutic outcomes in neurodegenerative diseases. The structural, chemical, and biological versatility of key polysaccharides control their ability to enhance therapeutic efficacy through nanoparticle‐based formulations. By tailoring these nanomaterials for immune modulation and neuroprotection, their potential to address both the inflammatory and neurodegenerative aspects of CNS disorders is enhanced. Furthermore, the fine‐tuning of polysaccharide‐based nanoparticles through combination with other polymers or active compounds can lead to the development of innovative biomaterials with enhanced properties, which are poised to revolutionize the treatment landscape for neurodegenerative diseases.

In conclusion, polysaccharide‐based nanoparticles represent a promising frontier in the development of immunomodulatory therapies for CNS disorders, especially for neurodegenerative diseases. Their capacity to mitigate neuroinflammation, promote neuronal regeneration, and provide targeted delivery of therapeutic agents positions them as key tools in advancing the treatment of AD, PD, and MS. Future research should continue to explore the full potential of these materials, optimizing their design and functionality to further enhance their clinical efficacy and impact in the field of neurodegenerative disease treatment.

## AUTHOR CONTRIBUTIONS

Leto‐Aikaterini Tziveleka, Mariafrancesca Cascione, and Valeria De Matteis conceptualized this review, and wrote, revised and edited the manuscript. Leto‐Aikaterini Tziveleka, Paolo Pellegrino, Annalisa Bianco, and Stefano Leporatti wrote the original draft. All authors read and approved the final content of this manuscript.

## CONFLICT OF INTEREST STATEMENT

The authors declare no conflicts of interest.

## ETHICS STATEMENT

Not applicable.

## DECLARATION ON THE USE OF AI

The authors declare that they use AI (ChatGPT 4.0) to improve the English language and enhance the readability of the manuscript.

## Data Availability

Not applicable as no data were generated or analyzed in this review.
